# Transcriptomic evidence for immaturity of the prefrontal cortex in patients with schizophrenia

**DOI:** 10.1186/1756-6606-7-41

**Published:** 2014-05-29

**Authors:** Hideo Hagihara, Koji Ohira, Keizo Takao, Tsuyoshi Miyakawa

**Affiliations:** 1 Division of Systems Medical Science, Institute for Comprehensive Medical Science, Fujita Health University, 1-98 Dengakugakubo, Kutsukake-cho, Toyoake, Aichi 470-1192, Japan; 2 CREST, JST, 4-1-8 Honcho, Kawaguchi, Saitama 332-0012, Japan; 3 Section of Behavior Patterns, Center for Genetic Analysis of Behavior, National Institute for Physiological Sciences, 38 Aza-Nishigo-naka, Myodaiji-cho, Okazaki, Aichi 444-8787, Japan

**Keywords:** Schizophrenia, Transcriptome, Prefrontal cortex, Immaturity, Parvalbumin, Endophenotype

## Abstract

**Background:**

Schizophrenia, a severe psychiatric disorder, has a lifetime prevalence of 1%. The exact mechanisms underlying this disorder remain unknown, though theories abound. Recent studies suggest that particular cell types and biological processes in the schizophrenic cortex have a pseudo-immature status in which the molecular properties partially resemble those in the normal immature brain. However, genome-wide gene expression patterns in the brains of patients with schizophrenia and those of normal infants have not been directly compared. Here, we show that the gene expression patterns in the schizophrenic prefrontal cortex (PFC) resemble those in the juvenile PFC.

**Results:**

We conducted a gene expression meta-analysis in which, using microarray data derived from different studies, altered expression patterns in the dorsolateral PFC (DLFC) of patients with schizophrenia were compared with those in the DLFC of developing normal human brains, revealing a striking similarity. The results were replicated in a second DLFC data set and a medial PFC (MFC) data set. We also found that about half of the genes representing the transcriptomic immaturity of the schizophrenic PFC were developmentally regulated in fast-spiking interneurons, astrocytes, and oligodendrocytes. Furthermore, to test whether medications, which often confound the results of postmortem analyses, affect on the juvenile-like gene expressions in the schizophrenic PFC, we compared the gene expression patterns showing transcriptomic immaturity in the schizophrenic PFC with those in the PFC of rodents treated with antipsychotic drugs. The results showed no apparent similarities between the two conditions, suggesting that the juvenile-like gene expression patterns observed in the schizophrenic PFC could not be accounted for by medication effects. Moreover, the developing human PFC showed a gene expression pattern similar to that of the PFC of naive Schnurri-2 knockout mice, an animal model of schizophrenia with good face and construct validity. This result also supports the idea that the transcriptomic immaturity of the schizophrenic PFC is not due to medication effects.

**Conclusions:**

Collectively, our results provide evidence that pseudo-immaturity of the PFC resembling juvenile PFC may be an endophenotype of schizophrenia.

## Background

Schizophrenia is a devastating and complex brain disorder. Although many susceptibility genes have been identified through genome-wide association studies [1-4], each gene exerts only a small to moderate effect on overall disease risk. Identifying endophenotypes in the brains of patients with schizophrenia is now considered the way to understand the etiology and mechanisms of the disorder. Growing evidence from postmortem [5-9] and animal studies [10-12] implicates abnormal neurodevelopment in the pathogenesis of schizophrenia and other psychiatric disorders. We screened >160 mutant mouse strains using a large-scale comprehensive behavioral test battery and identified several strains with behavioral traits corresponding to schizophrenia [13]. We examined the brains of the latter group using various approaches and found abnormalities in the dentate gyrus (DG) of the hippocampus in these mutants. That is, the molecular and electrophysiological features of DG neurons in the adult hippocampus of these mouse strains showed similarities to those of immature DG neurons in normal mice, a phenomenon termed the “immature DG” (iDG). To date, identified mouse strains with an iDG phenotype include the alpha-calcium/calmodulin-dependent protein kinase II heterozygous knockout (HKO) [12], schnurri-2 (Shn-2) KO [11], and mutated synaptosomal-associated protein 25 (SNAP-25) knock-in mice [10]. Importantly, postmortem analysis revealed an iDG-like signature in the brains of patients with schizophrenia or bipolar disorder [8]. We therefore proposed that the iDG is a potential endophenotype of several psychiatric disorders, including schizophrenia and bipolar disorder. The maturation of gamma-aminobutyric acid (GABA) signaling, characterized by progressive developmental switches in expression from GAD25 to GAD67 and from NKCC1 to KCC2, is abnormal in the hippocampus of patients with schizophrenia [6]. In addition to the hippocampus, we found abnormal development and maturation in the cortex in a mouse model of schizophrenia [11]. Abnormalities in development and maturation have been also implicated in the cortex of patients with schizophrenia. Risk alleles for schizophrenia may directly affect PFC development [14,15]. Torkamani *et al*. showed that the age-related decline of genes associated with developmental processes, such as neuronal differentiation, neurite outgrowth, and synaptic transmission, appeared to be slowed in the cortex of patients with schizophrenia [7]. This suggests that the expression of a subset of genes in the schizophrenic brain has become arrested at an adolescent (up to 19 years) developmental stage [16]. Consistent with this idea, a hyperdopaminergic state in patients with schizophrenia has been suggested to resemble the dopamine hyperactivity in the adolescent brain [17]. In the case of the GABA_A_ receptor, α1 subunit expression increases during PFC development and persists into adulthood, whereas α2 subunit expression decreases [18,19]. A decrease in α1 subunits [18,20,21] and an increase in α2 subunits [21,22] have been found in the schizophrenic PFC. Changes in GABA_A_ receptor subunits in schizophrenia may reflect a cortex held in a state of immaturity during adulthood. Furthermore, fast-spiking interneurons (FS neurons) in the cortices of patients with schizophrenia show maturational abnormalities [5]. Several lines of evidence show that expression levels of parvalbumin (PV), a marker of FS neurons, are decreased in the PFC of these patients [23-28]. PV immunoreactivity first appears in the PFC around 3–6 months of age and PV mRNA increases 20-fold from the neonatal stage to the adulthood [28,29], indicating that PV is a marker for mature FS neurons. The FS neurons in the cortices of patients with schizophrenia were hypothesized to be immature [5]. It was recently shown that, in schizophrenia, this neuron type retains a pseudo-immature status with regard to gene expression profiles [5]. Shn-2 KO mice, a mouse model of schizophrenia with iDG in the hippocampus, showed a decrease in the number of PV-positive cells in the frontal cortex, without signs of neurodegeneration in either region [11], which suggests that the immature signature can be seen in the mutant mice not only in the hippocampus but also in the frontal cortex. In contrast, somatostatin expression, a marker for a certain interneuron type, decreases from birth in the normal PFC, but shows a significant decrease in the schizophrenic PFC [28,30]. Considering that there are genes with expression patterns that are inconsistent with an immature phenotype in the schizophrenic PFC, it is important to evaluate immaturity in the brain using genome-wide gene expression profiles. However, genome-wide gene expression patterns in the brains of patients with schizophrenia and those of normal infants have not been directly compared.

Reports on studies of large-scale gene expression in various regions of the schizophrenic brain, including PFC, have been accumulating in publicly available databases. In this study, we performed bioinformatics analyses of such data (public microarray data sets) to see if the maturational state of the PFC is affected in schizophrenia. We compared genome-wide gene expression patterns of human developing PFC and adult schizophrenic PFC using different data sets reported independently.

## Results

### Gene expression patterns in the PFC of patients with schizophrenia resembling those in developing normal infants

**Figure 1:**
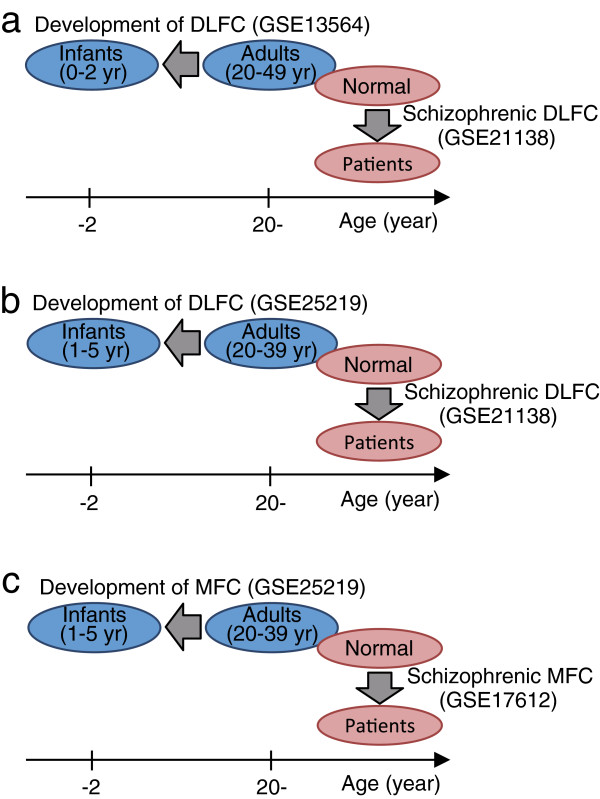
**Comparison of gene expression patterns between the developing and adult schizophrenic prefrontal cortex (PFC). (a)** The gene expression pattern in the dorsolateral frontal cortex (DLFC; Brodmann area [BA]46) of patients with schizophrenia (GSE21138, patients [26.1 ± 2.05 years] compared with controls [28.8 ± 2.55 years]) was compared with that in the DLFC (BA46) of normal infants (GSE13564, infants <2 years, compared to adults, 20–49 years). **(b)** The gene expression pattern in the DLFC (BA46) of patients with schizophrenia (GSE21138, patients compared with controls) was compared with that in the DLFC (BA9 and 46) of normal infants (GSE25219, infants, 1–5 years, compared with adults, 20–39 years). Note that microarray data sets of the normal developing DLFC were obtained from two independent research groups and were used in **(a)** and **(b)**, respectively. **(c)** The gene expression pattern in the medial prefrontal cortex, (MFC) (BA10) of patients with schizophrenia (GSE17612, patients [73.3 ± 15.2 years] compared with controls [69.0 ± 21.6 years]) was compared with that in the MFC (BA24, 32, 33) of normal infants (GSE25219, infants, 1–5 years, compared with adults, 20–39 years).

**Figure 2:**
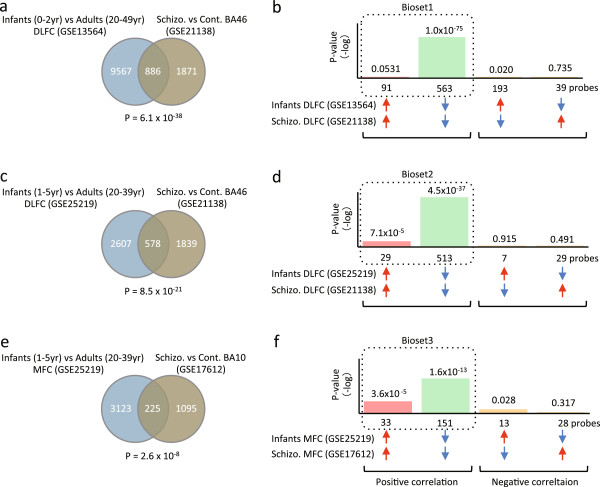
**Transcriptional immaturity of PFC in the schizophrenic brain. (a, c, e)** Venn diagrams illustrating the overlap in transcriptome-wide gene expression changes in the DLFC (BA46) of patients with schizophrenia (patients compared with controls) and normal infants (infants <2 years, compared with adults, 20–49 years) **(a)**, the DLFC (BA46) of patients with schizophrenia (patients compared with controls) and that in the DLFC (BA9 and 46) of normal infants (infants, 1–5 years, compared with adults, 20–39 years) **(c)**, and the MFC (BA10) of patients with schizophrenia (patients compared with controls) and that in the MFC (BA24, 32, 33) of normal infants (infants, 1–5 years, compared with adults, 20–39 years) **(e)**. **(b, d, f)***P*-values of overlap between the schizophrenic DLFC and normal developing DLFC (<2 years compared with 20–49 years) data sets **(b)**, schizophrenic DLFC and normal developing DLFC (1–5 years compared with 20–39 years) data sets **(d)**, and schizophrenic MFC and normal developing MFC (1–5 years, compared with 20–39 years) data sets **(f)**. Bar graphs illustrate the *P*-values of overlaps of genes upregulated (*red arrows*) or downregulated (*blue arrows*) by each condition, between the two conditions. Bonferroni correction was used to adjust the significant level by the number of pairs of datasets included in each study (see the Methods section and Additional file 1: Table S27). The genes that showed the same directional changes in expression, or positive correlation, between two groups in **(b)**, **(d)**, and **(f)** were designated Bioset1–3 (surrounded by dotted line), respectively. These Biosets were used in the analyses for pathway enrichment (Additional file 1: Table S6, S7, S9) and cell-type contribution (Figure 3).

To assess whether, or to what extent, the overall gene expression profile in the schizophrenic PFC is similar to that in the developing PFC, we comparatively analyzed human microarray data sets as follows: development of the normal dorsolateral frontal cortex (DLFC; GSE13564; Brodmann’s area [BA]46) compared to the schizophrenic DLFC (short-DOI subgroup in GSE21138; BA46; Figure 1a). Microarray data sets were compared using non-parametric rank-based statistical methods, which incorporates information regarding rank and the direction of gene expression changes into overlap *P*-values (see Methods section for details). This schizophrenic DLFC data was obtained by querying the NextBio database for the curated microarray data in humans that show gene expression patterns similar to those for the developing DLFC (<2 years vs. 20–49 years; GSE13564 [31]). We identified a high overlap in *P*-values between the schizophrenic DLFC (short-DOI vs. age-matched control; GSE21138 [32]) and the normally developing DLFC (*P =* 6.1 × 10^-38^, Additional file 1: Table S1). In addition, we found the intermediate-DOI (*P =* 1.1 × 10^-16^) and long-DOI (*P* = 0.0002) subgroups showed less overlap in *P*-values when compared with the normally developing DLFC (Additional file 1: Table S2). Therefore, we conducted further comparative analyses with the data from the short-DOI subgroup. By comparing these microarray data sets on the developing and adult schizophrenic DLFC, we found a high degree of gene expression overlap between them (Figure 2a). Out of 2757 probes altered in the schizophrenic DLFC, 886 were shared with probes altered during the DLFC development, indicating statistically significant similarities in the transcriptome change between them (overlap *P*-value: *P =* 6.1 × 10^-38^, Figure 2a, Additional file 1: Table S3). Among the 886 probes, 91 were upregulated (*P =* 0.053) and 563 were downregulated in both groups (*P =* 1.0 × 10^-75^; Figure 2b). These 91 and 563 probes with the same direction of change in expression between the two groups are denoted as showing a positive correlation. We also found that 193 probes were upregulated in the developing DLFC and downregulated in schizophrenia (*P =* 0.197), and 39 probes were downregulated in the developing DLFC and upregulated in schizophrenia (*P =* 0.735; Figure 2b). The probes that showed opposite changes between the two groups are denoted as showing a negative correlation. The number of probes with a positive correlation (654 out of 886 probes) was significantly higher than expected on the basis of chance (χ^2^(1) = 201.00, *P =* 1.27 × 10^-45^; Figure 2b). Collectively, these findings indicate the gene expression patterns in the schizophrenic DLFC are highly similar to those in the infant DLFC.

To confirm further the results obtained in the schizophrenic DLFC, we repeated the comparative analysis using different microarray data examining developmentally regulated gene expression in DLFC (Figure 1b). These microarray data were obtained from a differential analysis of infants (1–5 years) and adults (20–39 years; GSE25219 [33]). We found that a large number of genes overlapped in this comparison as well (578 probes, *P =* 8.5 × 10^-21^, Figure 2c, Additional file 1: Table S4). Among the 578 overlapped probes, 542 showed a positive correlation: 29 (*P =* 7.1 × 10^-5^) and 513 (*P =* 4.5 × 10^-37^) were upregulated and downregulated in both groups, respectively (Figure 2d). Seven probes were upregulated in the developing DLFC but downregulated in schizophrenia (*P =* 0.915), and 29 probes were downregulated in the developing DLFC but upregulated in schizophrenia (*P =* 0.491; 36 probes with negative correlation; Figure 2d). The number of probes with a positive correlation (542 out of 578 probes) was significantly higher than expected (χ^2^(1) = 442.97, *P =* 2.45 × 10^-98^; Figure 2d). These findings also indicate that gene expression patterns in the schizophrenic DLFC are highly similar to those in the infant DLFC.

To further confirm the transcriptomic immaturity in the DLFC of patients with schizophrenia, we examined gene expression patterns in data sets examined in other microarray studies. When the gene expression patterns in the developing DLFC (infants vs. adults; GSE11512 [34]) were compared with those in the 2 schizophrenia cohorts (patients vs. controls; GSE53987 or GSE12649 [35]), we found statistically significant similarities in both comparisons (*P* = 8.4 × 10^-22^ and *P* = 1.8 × 10^-5^; Additional file 2: Figure S1). Overall, essentially the same results as those shown in Figure 2a and c were obtained when using the data sets from the developing DLFC and schizophrenic DLFC. The result of a high similarity of gene expression between developing normal DLFC and adult schizophrenic DLFC was replicated by comparing the schizophrenic DLFC data with different data for the developing DLFC.

We also tested for similarity of gene expression patterns in another part of the frontal lobe using microarray data on the developing normal MFC and adult schizophrenic MFC (Figure 1c). When we used a NextBio analysis to compare the gene expression patterns in the schizophrenic MFC (GSE17612 [36]) with those in the developing normal MFC (GSE25219 [33]), we found statistically significant similarities in the transcriptome change patterns between the data sets (*P =* 2.6 × 10^-8^, Figure 2e, Additional file 1: Table S5). Out of the 225 probes overlapped between the 2 groups (Figure 2e, Additional file 1: Table S5), 184 showed a positive correlation within 2 groups, which was significantly higher than expected (χ^2^(1) = 90.88, *P =* 1.52 × 10^-21^, Figure 2f). This also indicated that the gene expression patterns in the schizophrenic MFC are similar to those in the infant MFC. The subjects chosen by Maycox *et al.* had an average age of 70 years [36]. In a comparative analysis using data from subjects that were >60 years old as an adult group in the developmental data set, we also found similarity between the two data sets (*P* = 0.0053, Additional file 3: Figure S2). Considering this result and the data shown in Figure 2, the gene expression pattern in the MFC of patients with schizophrenia may be similar to that of infants.

**Figure 3:**
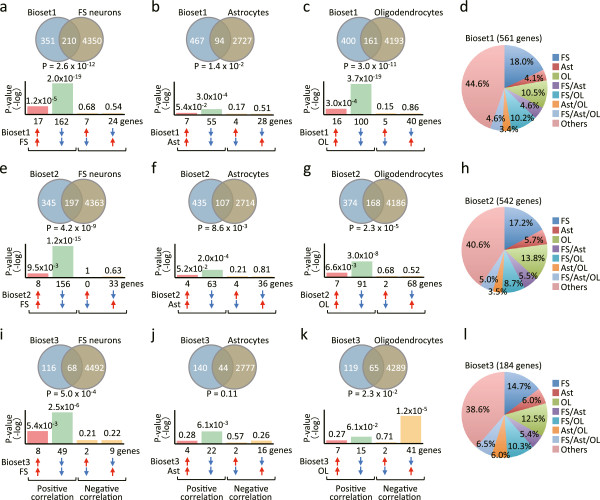
**Cell-type contributions to transcriptional immaturity in schizophrenic PFC.** Genes showing the same directional changes in expression between the normal developing and adult schizophrenic PFC (**a-d**, Bioset1 in DLFC data set; **e-h**, Bioset2 in second DLFC data set; **i-l**, Bioset3 in MFC data set) were compared to those obtained from cell-type specific developmental experiments (**a**, **e**, **i**, FS neurons [GSE17806]; **b**, **f**, **j**, astrocytes [GSE9566]; **c**, **g**, **k**, oligodendrocytes [GSE9566]). Venn diagrams illustrate the overlap in transcriptome-wide gene expression changes between two conditions. Bonferroni correction was used to adjust the significant level by the number of pairs of datasets included in each study (see the Methods section and Additional file 1: Table S27). Bar graphs illustrate the *P*-values of overlaps of genes upregulated (*red arrows*) or downregulated (*blue arrows*) by each condition, between the two conditions. Note that the scale of the y-axis is the same in **a–c**, **e–g**, and **i–k. (d, h, l)** Pie chart representing the percentage that each cell-type contributes to altered gene expression in Bioset1 **(d)**, Bioset2 **(h)**, and Bioset3 **(l)**. Ast, astrocytes; FS, FS neurons; OL, oligodendrocytes.

Taken together, the results of the analyses in DLFC and MFC showed similar trends, providing evidence that whole-tissue gene expression patterns in the PFC of patients with schizophrenia resemble those of infants. The genes showing positive correlations in the two analyses for DLFC (Figure 2b, d) and in the analysis for MFC (Figure 2f) were designated Bioset1–3, respectively. These Biosets were used in the pathway analysis (Additional file 1: Table S6, S7, S9) and cell-type contribution analysis (Figure 3).

### Enriched pathways in juvenile-like PFC of patients with schizophrenia

We analyzed the gene list (all genes in Biosets 1–3) for enrichment of gene ontology (GO) and canonical pathways using DAVID bioinformatics databases (Additional file 1: Table S6). Significantly enriched (Benjamini-adjusted *P <* 0.05) GO categories and KEGG pathways for genes that changed in both the developing normal PFC and adult schizophrenic PFC with a positive correlation (Biosets 1–3) are presented in Additional file 1: Table S6. In addition, we examined the enriched pathways in each Bioset using NextBio (Additional file 1: Table S7). The top 30 enriched pathways are listed in Additional file 1: Table S7, ranked according to their enrichment score; the score is based on the overall statistical significance and consistency of the enrichment, or overlap, between the genes that make up the pathway involved and each Bioset queried. In analyses using both DAVID and NextBio, we found statistically significant enrichments for pathways related to mitochondrial components, vacuolar components, and bacterial infection in these Biosets (Additional file 1: Table S6, S7). A pathway analysis using DAVID (without multiple comparison correction) for the 22 transcripts, which overlapped among three Biosets (Additional file 4: Figure S3, Additional file 1: Table S8), showed enrichments in pathways related to energy metabolism (Additional file 1: Table S9).

### Cell type contributions to transcriptional immaturity of PFC in patients with schizophrenia

Next, we determined which cell types contribute to the juvenile-like gene expression patterns in the schizophrenic PFC. Because there is no human microarray data on the development of specific cell types, we used mouse data on the development of FS neurons [37], astrocytes, and oligodendrocytes [38] for assessing cell type contributions. We estimated the contribution of each cell type by comparing 2 groups: genes altered in both the normal developing human PFC and adult schizophrenic PFC with the same directional change in expression, and those developmentally regulated in particular cell types.

The microarray data on FS neurons were obtained from GFP-expressing neurons isolated by cell sorter from transgenic mice expressing GFP under the control of the *GAD1* promoter at postnatal days (P) 7–40 [37]. The transgenic mice were engineered by the genomic incorporation of a 200 kb GAD1 bacterial artificial chromosome fused to a GFP coding sequence. The combination of the promoter and position of integration effects restricted GFP expression to a homogenous subset of GABAergic FS neurons. *GAD1* promoter-driven GFP expression is a stable marker for prospective FS PV-positive cortical interneurons during development in this mouse line [37]. Data from P40 and P7 of the FS neurons were compared, and the result was taken as a sample of the developmental gene expression changes of this cell type in this study (Table 1). GFP-positive astrocytes, which were collected by cell sorter from S100β (a marker of astrocyte)-GFP transgenic mice at P1–30, were processed for microarray analysis [38], and we used these data to sample the developmental gene expression changes of astrocytes by comparing P17–30 with P1–8 (Table 1). The oligodendrocytes were purified by antibody-based panning methods using antibodies against maturational markers (PDGFRα, GalC, and MOG) expressed by mice at P16, and were then processed for microarray analysis [38]. We compared the microarray data from MOG-positive myelinating oligodendrocytes with GalC-positive premyelinating oligodendrocytes, and used the result as a sample of the developmental gene expression changes of oligodendrocytes. The differences in the methods used for cell collection and in the ranges of maturational time points of each cell type should be taken into account when directly comparing the extent of contribution among these cell types.

**Table 1:** Microarray data used in this study

***GEO accession***	***Reference***	***Microarray platform***	***Samples***	***No. of samples***
GSE13564	Harris *et al.*, 2009 [31]	GPL570 (HG-U133 Plus 2.0)	Dorsolateral prefrontal cortex (BA46) of infants (0-2 yr) and adults (20-49 yr)	Infants = 18, adults = 13
GSE25219	Kang *et al.*, 2011 [33]	GPL5175 (HuEx-1.0st)	Dorsolateral prefrontal cortex (BA9, 46) of infants (1-5 yr) and adults (20-39 yr); medial prefrontal cortex (BA24, 32, 33) of infants (1-5 yr) and adults (20-39 yr); superior temporal cortex of infants (0-5 yr) and adults (20-39 yr)	DLFC: infants =6, adults =14; MFC: infants = 5, adults = 14; STC: infaints =7, adults = 13
GSE11512	Somel *et al.*, 2009 [34]	GPL6879 (HG-U133 Plus 2.0)	Dorsolateral prefrontal cortex of infants (0.1-0.3 yr) and adults (20-49 yr)	Infants = 7, adults = 11
GSE37721	Sterner *et al.*, 2012 [94]	GPL6947 (Illumina HumanHT-12 V3.0)	Superior temporal cortex of infants (0-6 yr) and adults (24-48 yr)	Infants = 7, adults = 5
GSE21138	Narayan *et al.*, 2008 [32]	GPL570 (HG-U133 Plus 2.0)	Prefrontal cortex (BA46) of schizophrenia (26.1 ± 2.1 yr) and control (28.8 ± 2.6 yr) subjects	SZ = 8, CTL = 7
GSE12649	Iwamoto *et al.*, 2008 [35]	GPL96 (HG-U133A)	Prefrontal cortex (BA46) of schizophrenia (42.6 ± 8.5 yr) and control (44.1 ± 7.7 yr) subjects	SZ = 35, CTL = 34
GSE53987	Lanz *et al.*, 2014	GPL570 (HG-U133 Plus 2.0)	Prefrontal cortex (BA46) of schizophrenia (46.0 ± 8.6 yr) and control (48.1 ± 7.6 yr) subjects	SZ = 15, CTL = 19
GSE17612	Maycox *et al.*, 2009 [36]	GPL570 (HG-U133 Plus 2.0)	Anterior prefrontal cortex (BA10) of schizophrenia (73.3 ± 15.2 yr) and control (69.0 ± 21.6 yr) subjects	SZ = 26, CTL =21
GSE21935	Barnes *et al.*, 2011 [95]	GPL570 (HG-U133 Plus 2.0)	Superior temporal cortex (BA22) of schizophrenia (72.2 ± 16.9 yr) and control (67.7 ± 22.2 yr) subjects	SZ = 23, CTL = 19
GSE17806	Okaty *et al.*, 2009 [37]	Mouse430.2.0	Fast-spiking interneurons isolated from somatosensory cortex of P7 and P40 mice	P7 = 3, P40 = 3
GSE9566	Cahoy *et al.*, 2008 [38]	Mouse430.2.0	Astrocytes isolated from forebrain of P1-30 mice and oligodendrocytes isolated based on maturation markers expression from forebrain of P16 mice	Ast: P1-8 = 4, P17-30 = 4; OL: OL = 4, Myelin OL = 4
GSE4675	Semeralul *et al*., 2006 [96]	Mouse430A.2.0	Whole frontal cortex of 2 and 10 weeks old mice	2 weeks old = 6, 10 weeks old = 4
GSE42775	Takao *et al.*, 2013 [11]	Mouse430.2.0	Medial frontal cortex of Schnurri-2 KO and wild-type mice	KO = 6, WT = 6
GSE45229	Kondo *et al.*, 2013 [97]	Mouse430.20	Frontal cortex of mice treated with haloperidol, quetiapine, or a vehicle	Haloperidol (0.3 mg/kg) = 4; Haloperidol (1 mg/kg) = 4; Quetiapine (10 mg/kg) = 4; Quetiapine (100 mg/kg) = 4; Vehicle = 4
GSE2547	Fatemi *et al.*, 2006 [98]	Rat430.2.0	Frontal cortex of rats treated with olanzapine or vehicle	Olanzapine (2 mg/kg) = 20, vehicle = 20

Of 561 genes in Bioset1, which consists of genes with expression changes in both human developing DLFC and schizophrenic DLFC with a positive correlation, 210 genes (37.4%) were shared with data sets reflecting mouse FS neuron development (overlap *P*-value: *P =* 2.6 × 10^-12^; Figure 3a, Additional file 1: Table S10). Furthermore, it was found that 94 genes (16.8%) and 161 genes (28.7%) were overlapped between Bioset1 and data sets of astrocyte development (*P =* 0.014; Figure 3b, Additional file 1: Table S11) and oligodendrocyte development (*P =* 3.0 × 10^-11^; Figure 3c, Additional file 1: Table S12), respectively. Importantly, most of the genes commonly changed in Bioset1 and in the development of each cell type showed the same directional change in expression: a positive correlation (179 out of 210 genes in comparison with FS neuron development, χ^2^(1) = 104.30, *P =* 1.73 × 10^-24^, Figure 3a; 62 out of 94 genes in astrocytes, χ^2^(1) = 9.57, *P =* 1.97 × 10^-3^, Figure 3b; and 116 out of 161 genes in oligodendrocytes, χ^2^(1) = 31.31, *P =* 2.20 × 10^-8^, Figure 3c). In addition, among these 561 genes, 101 (18.0%), 23 (4.1%) or 59 genes (10.5%) showed significant changes developmentally only in mouse FS neurons, astrocytes, or oligodendrocytes, respectively (Figure 3d, Additional file 1: Table S13). Twenty-six (4.6%), 57 (10.2%), and 19 genes (3.4%) were shared between FS neurons and astrocytes, FS neurons and oligodendrocytes, and astrocytes and oligodendrocytes, respectively. Twenty-six (4.6%) out of 561 genes in Bioset1 showed developmental regulation commonly in these three cell types (Figure 3d). On the whole, 311 out of 561 genes (55.4%), representing juvenile-like expression patterns in schizophrenic DLFC, could be explained as representing transcriptional immaturity in just these three cell types (Figure 3d).

Furthermore, we observed similar trends in the results regarding cell-type contributions to the juvenile-like gene expressions in Bioset2 (a second data set for DLFC; Figure 3e–h) and in Bioset3 (a data set for MFC; Figure 3i–l). In comparisons using Bioset2, the expression of 197, 107, and 168 genes were overlapped with data sets from FS neuron development (*P =* 4.2 × 10^-9^, Figure 3e, Additional file 1: Table S14), astrocyte development (*P =* 0.0086, Figure 3f, Additional file 1: Table S15), and oligodendrocyte development (*P =* 2.3 × 10^-5^, Figure 3g, Additional file 1: Table S16), respectively. The expression of 164 out of 197 genes (χ^2^(1) = 87.11, *P =* 1.03 × 10^-20^, Figure 3e), 67 out of 107 genes (χ^2^(1) = 6.81, *P =* 0.0091, Figure 3f), and 98 out of 168 gens (χ^2^(1) = 4.67, *P =* 0.031, Figure 3g) showed a positive correlation in each comparison. In comparisons with Bioset3, the expression of 68, 44, and 65 genes were overlapped in data sets from FS neuron (*P =* 5.0 × 10^-4^, Figure 3i, Additional file 1: Table S18), astrocyte (*P =* 0.107, Figure 3j, Additional file 1: Table S19), and oligodendrocyte development (*P =* 0.023, Figure 3i, Additional file 1: Table S20), respectively. The expression of 57 out of 68 genes (χ^2^(1) = 31.12, *P =* 2.43 × 10^-8^, Figure 3i) and 26 out of 44 genes (χ^2^(1) = 1.45, *P =* 0.228, Figure 3j) showed positive correlations in comparisons with FS neuron and astrocyte development, respectively. In contrast, 43 out of 65 genes showed negative correlations in comparison with oligodendrocyte development (χ^2^(1) = 6.78, *P =* 0.0092, Figure 3k), suggesting putatively elevated maturation of oligodendrocytes in the MFC of patients with schizophrenia.

### Transcriptional immaturity of PFC in an animal model of schizophrenia but not in rodents treated with antipsychotics

Most patients diagnosed with schizophrenia are medicated with antipsychotics. Consequently, juvenile-like gene expression patterns observed in the schizophrenic PFC could result from medications. We therefore tested whether antipsychotic treatments induce transcriptomic immaturity in PFC by comparing gene expression patterns in the PFC of rodent treated with antipsychotics (haloperidol, quetiapine [GSE45229], or olanzapine [GSE2547]) with those of human developing PFC. The analyses revealed no apparent similarities between the given two conditions, except for a comparison between olanzapine-treated rodent PFC and the human developing MFC (Additional file 5: Figure S4). Similarity between the olanzapine-treated rodent PFC and the human developing MFC was weakly but marginally significant after Bonferroni correction (significance level of *P* < 0.0125; Additional file 5: Figure S4o; see the Methods section and Additional file 1: Table S27), suggesting that olanzapine may have some effect on the induction of juvenile-like gene expression in adult PFC.

**Figure 4:**
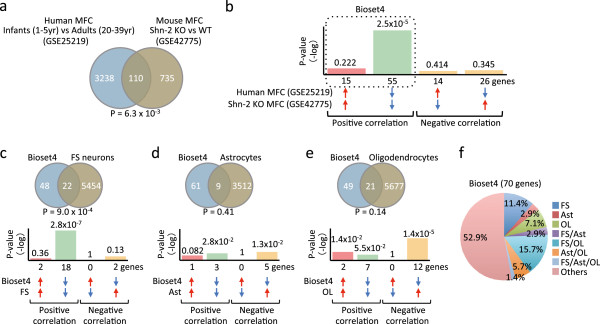
**Transcriptional immaturity of PFC in an animal model of schizophrenia. (a)** Venn diagram illustrating the overlap in transcriptome-wide gene expression changes in the MFC of Shn-2 KO mice (Shn-2 KO compared to wild type) and that in human infants (GSE25219; infants, 1–5 years, compared with adults, 20–39 years). **(b)***P*-values of overlap between Shn-2 KO mouse and human infant data sets. Bar graph illustrates the *P*-values of overlaps of genes upregulated (*red arrows*) or downregulated (*blue arrows*) by each condition, between the two conditions. The genes that showed the same directional changes in expression, or positive correlation, between two groups were designated Bioset4. **(c–e)** Comparison of gene expression derived from cell-type specific developmental experiments (**c**, FS neurons [GSE17806]; **(d)**, astrocytes [GSE9566]; **(e)**, oligodendrocytes [GSE9566]) with Bioset4. Bonferroni correction was used to adjust the significant level by the number of pairs of datasets included in each study (see the Methods section and Additional file 1: Table S27). **(f)** Pie chart representing the percentage that a particular cell type contributes to the altered gene expression pattern in Bioset4.

Furthermore, we tested a mouse strain that models schizophrenia, that had no history of drug treatment, for evidence of immaturity in the PFC. The disease-associated part of the gene expression pattern in the MFC of the mouse schizophrenia model, Shn-2 KO mice (deduced as Shn-2 KO compared with wild-type, GSE42775 [11]), was compared with the developmentally-related part of the expression pattern deduced to exist in the MFC (BA24, 32, and 33) of human infants (infants, 1–5 years, compared with adults, 20–39 years, GSE25219 [33]). Shn-2, a nuclear factor-κB site-binding protein, binds to the enhancers of major histocompatibility complex class I genes and inflammatory cytokines, which harbor common variant single-nucleotide polymorphisms associated with schizophrenia [3,39,40]. Shn-2 KO mice display behavioral abnormalities that resemble the symptoms of human schizophrenia, including working memory deficits and decreased social behaviors [11]. Moreover, the brains of Shn-2 KO mice have numerous schizophrenia-related phenotypes, such as transcriptome/proteome changes similar to those of postmortem patients with schizophrenia, decreased PV and GAD67 levels, increased theta and decreased gamma power on electroencephalograms, and a thinner cortex [11,41-44]. Thus, Shn-2 KO mice serve as an animal model of schizophrenia with good face and construct validity. Recently, de novo damaging mutations were detected in the schizophrenic PFC in the gene encoding Shn-1 (also called Hivep1), another member of the Schnurri family, which was suggested to be involved in pathways important for brain development [14]. Using microarray data on MFC of Shn-2 KO mice, we investigated whether the mouse model of schizophrenia has transcriptional immaturity in the PFC. Both MFC of human and MFC of rodents receive projections from the medial part of the mediodorsal nucleus of the thalamus [45], which suggests the mouse MFC is the counterpart of the human MFC. One hundred and 10 genes overlapped between human developing MFC and Shn-2 KO mice MFC, indicating significant similarities in the pattern of transcriptome changes between the two groups (*P =* 0.0063, Figure 4a, Additional file 1: Table S22). Of those genes, 70 showed the same directional changes in expression (Figure 4b), and of these genes, 55 were downregulated (*P =* 2.5 × 10^-5^) whereas 15 were upregulated (*P =* 0.222). To determine what cell type contributes to this transcriptome change, 70 genes that showed a positive correlation (Bioset4) were compared with data sets from cell type-specific developmental experiments (Figure 4c-e). The expression of 22 genes was changed in common between Bioset4 and the data set for FS neuron development (*P =* 9.0 × 10^-4^, Figure 4c, Additional file 1: Table S23). Between Bioset4 and the data set for astrocyte development, only nine genes changed in common (*P =* 0.411, Figure 4d, Additional file 1: Table S24). Although 21 genes were differentially expressed in parallel between Bioset4 and the oligodendrocytes development data set (*P =* 0.136, Figure 4e, Additional file 1: Table S25), more than half of those genes were regulated in the opposite direction. Of the 70 genes in Bioset4, 20 (28.6%) were regulated similarly in immature FS neurons (Figure 4f, Additional file 1: Table S26). These results suggest that the gene expression pattern of FS neurons in the PFC of Shn-2 KO mice resembles that of immature FS neurons.

## Discussion

In this study, we show that the schizophrenic PFC resembles the juvenile PFC with respect to transcriptome-wide gene expression profiles. We compared relative gene expression in the DLFC and MFC of patients with schizophrenia (patients compared with controls) with that in corresponding regions of the human developing PFC (infants compared with adults), and showed striking similarities between them. Moreover, we revealed that transcriptional immaturity could be seen in multiple cell types in the schizophrenic PFC, including FS neurons, astrocytes, and oligodendrocytes. Our findings support the idea that immaturity in the PFC could be an endophenotype of schizophrenia.

Regarding gene expression, our bioinformatics analyses revealed highly significant similarities in the relative gene expression patterns in the PFC between patients with schizophrenia (as compared to healthy controls) and infants (as compared to adults). More specifically, the expression level of a large number of genes that normally increases during human DLFC development was decreased in the DLFC of patients with schizophrenia (Figure 2b). In other words, these genes can be considered maturation markers whose expression is disturbed in the DLFC of patients with schizophrenia. The similarity in the maturation marker expression patterns and those of schizophrenia markers in adults was extraordinary significant, and the result was replicated in 3 further analyses using other two DLFC data sets and an MFC data set, respectively (Figure 2d, f, Additional file 2: Figure S1b). Genes for immaturity markers with expression that decreases with age in the normal PFC also were affected in the PFC of patients with schizophrenia, but to a lesser extent. Thus, the transcriptional immaturity of the schizophrenic PFC could be characterized as preferential downregulation of maturation marker gene expression. When the gene expression patterns in the developing DLFC (infants vs. adults; GSE11512 [34]) were compared with those in the 2 schizophrenia cohorts (patients vs. controls; GSE53987 or GSE12649 [35]), we found statistically significant similarities in both comparisons (*P* = 8.4 × 10^-22^ and *P* = 1.8 × 10^-5^; Additional file 2: Figure S1). The relatively low overlap *P*-value in Additional file 2: Figure S1c relative to other comparisons (Figure 2a, 2c, Additional file 2: Figure S1a) may be due to the small number of transcripts that were changed in the patients in the GSE12649 study [35]. Transcript expression changes numbering 2757 (806 upregulated and 1951 downregulated), 1163 (529 upregulated and 634 downregulated) and 163 (118 upregulated and 45 downregulated) were found in the studies GSE21138 [32], GSE53987, and GSE12649 [35], respectively. The differences in the number of transcripts detected in these studies are probably due to differences in the types of microarray chip that they used (Table 1). The small number of transcripts (45) downregulated in the patients in the GSE12649 study [35] may have caused the small number of maturation markers that downregulated in these patients (13 transcripts in Additional file 2: Figure S1d). Functionally, a deficit in working memory is common in patients with schizophrenia and has been attributed to PFC dysfunction [46]. When the developmental changes in working memory capacity were tested in normal subjects, 8- to 12-year-olds performed more poorly than adolescents or adults (age groups 13–17 and 18–25, respectively) [47]. Together with these previous results, our results suggest the PFC in patients with schizophrenia resembles the PFC in normal infants, both functionally and in gene expression patterns.

In the study by Maycox *et al*., the MFC analysis was performed for subjects with schizophrenia and controls who had an average age of 70 years. In the present study, we compared the gene expression changes in the MFC of elderly subjects with schizophrenia (patients vs. controls) with those in the developing MFC (infants vs. relatively young adults [20–39 years]; Figure 1C), which revealed significant similarities between the two groups (*P* = 2.6 × 10^-8^; Figure 2e). This result might indicate that the relative change in gene expression patterns that define transcriptomic infancy can also be seen in a cohort of elderly subjects with schizophrenia. We also found that gene expression patterns in the schizophrenic MFC are similar to those in the normal infant MFC as compared to elderly adult MFC (Additional file 3: Figure S2), suggesting that both the DLFC and MFC of patients with schizophrenia represent juvenile-like gene expression profiles. In addition, we found a significant similarity in the gene expression patterns of the superior temporal cortex (STC) between normal infants and patients with schizophrenia (Additional file 6: Figure S5). These findings suggest that juvenile-like gene expression profiles also can be found in brain regions other than the PFC in patients with schizophrenia.

Our present results also show that juvenile-like gene expression in the PFC of patients with schizophrenia could be due to immaturity in multiple cell types, including FS neurons, astrocytes, and oligodendrocytes. In the case of immaturity in FS neurons and oligodendrocytes, our results are consistent with those of previous studies analyzing molecular expression or structures in these cell types in schizophrenia [5,48]. Dysregulated gene expression in GABAergic neurons is one of the most robust findings in schizophrenia neuropathology. It is also a well-replicated finding that expression of PV, a marker for FS neurons, is decreased in the PFC of patients with schizophrenia [49]. Considering that PV expression appears in the PFC only postnatally and drastically increases with age [28,29], PV is thought to be a marker for mature FS neurons. Therefore, FS cells were hypothesized to be immature in schizophrenia [5]. In this context, Gandal *et al*. developed an FS cell maturation index to evaluate maturity of FS neurons in the cortices of patients with psychiatric disorders including schizophrenia, bipolar disorder, and autism [5]. Using time-course gene expression data from developing FS cells that were positively correlated with PV expression levels, Gandal *et al*. showed a reduction of the index in cortices of patients with schizophrenia, bipolar disorder, and autism. In addition, a decrease in perineuronal nets (PNNs) has been reported in the schizophrenic PFC [50]. Considering that PNNs are extracellular matrices predominantly enriched around mature FS neurons [51], their decrease may imply the presence of pseudo-immature FS neurons in schizophrenic cortices. These results suggest that FS neurons stay at partially immature state in the cortices of patients with schizophrenia, which is consistent with our results.

Possible contributions of myelin and oligodendrocyte dysfunction to schizophrenia also have been suggested by many postmortem studies of the human brain at molecular [48] and ultrastructural levels [52]. This well-documented evidence for dysmyelination seems consistent with our finding of immaturity in oligodendrocytes. Specific markers identify the different stages of oligodendrocytes maturation: PDGFRα in oligodendrocyte progenitor cells, GalC in premyelinating oligodendrocytes, and MOG in myelinating oligodendrocytes. We used the data set for gene expression changes between premyelinating and myelinating mouse oligodendrocytes, which were purified based on maturation marker expression, as an index for oligodendrocyte immaturity in the present study [38]. The gene expression changes during oligodendrocyte maturation showed a significant positive correlation with those shared by the human developing and schizophrenic DLFC, suggesting immaturity of oligodendrocytes in the DLFC of patients with schizophrenia.

In addition to the previous studies suggesting abnormal maturational status of FS neurons and oligodendrocytes in the schizophrenic brain, we showed that immaturity of astrocytes could also be seen in schizophrenic brains. Possible observations of changes in astrocyte densities in the cortices of schizophrenia patients are controversial; however, previous studies have shown the expression of several astrocyte-related genes is abnormal in these patients [52]. For example, increased expression of GLT-1, a major glutamate transporter, has been reported in the PFC of patients with schizophrenia [53]. Considering the expression of GLT-1 in rat astrocytes declines as they mature [54], the increased GLT-1 expression in the schizophrenic PFC may imply the existence of astrocytes that partially resemble those in the immature brain. Such immaturity would support the idea that astrocyte function is linked to the pathophysiology of schizophrenia.

One might expect that, in patients with schizophrenia, a decreased expression of a large number of maturation marker genes might reflect the loss of cells expressing those genes (i.e., PV in FS neurons and MOG in oligodendrocytes). In one study, there was no significant change in the number of PV-expressing cells in the PFC of patients with schizophrenia, whereas the PV expression per cell decreased when compared with controls [25]. The number of astrocytes between the schizophrenic and control PFC also were not significantly different [55-57], even though expression of astrocyte-related genes is altered in the cortex of patients with schizophrenia [52,58]. Analyses of oligodendrocytes suggest that subtle oligodendrocyte or myelin anomalies, such as myelin sheath damage and a decreased mitochondria density [59], may be more important than the changes in cell density associated with the pathophysiology of schizophrenia [60]. In the frontal cortex of Shn-2 KO mice, no obvious hallmarks of neurodegeneration, including cell death, cell swelling, protein deposition, or nuclear condensation, were observed with immunohistological or electron microscopic analyses. The number of PV-positive cells was significantly reduced in the frontal cortex of Shn-2 KO mice when compared with controls [11]. This suggests that the PV expression per cell decreased to undetectable levels in the mutant mice, which is similar to the pathology of schizophrenia in humans. Thus, in the cortex of patients with schizophrenia, a lower gene expression may reflect changes in gene expression in these cell types, rather than cell death.

Recent studies suggest that certain cell types in several brain regions of patients with schizophrenia may exhibit maturation abnormalities. By assessing the expression levels of maturational markers, Walton *et al.* showed that the dentate granule cells of the patients may be persistently in pseudo-immature state [8]. Expression of PV is decreased not only in the PFC but also in the hippocampus of patients with schizophrenia [61]. Furthermore, a decrease in PNNs has been reported in the entorhinal cortex and amygdala of patients [51,62]. The decreases in PV and PNNs imply that FS neurons in those brain regions stay at pseudo-immature state. Expression of KCC2, a K^+^-Cl^-^ cotransporter that plays a role in GABAergic neurotransmission, is decreased in the PFC and hippocampus of the patients [6,63]. Considering that KCC2 expression rises as brain development progresses [64], the decreases indicate a pseudo-immature state in a certain type of neuron in the PFC and the hippocampus. In addition, we observed transcriptomic immaturity in the superior temporal cortex (STC) of the patients (Additional file 6: Figure S5). These findings suggest that maturational abnormalities can be seen in certain cell types in several regions across the brain of patients with schizophrenia.

Although we showed that about half of the genes representing juvenile-like expression patterns in the schizophrenic PFC were developmentally regulated in three cell types (Figure 3d, h, l), there is also a possibility that these altered expression signals are partly due to maturational abnormalities in other cell types. Gene expression patterns representing transcriptomic immaturity in the schizophrenic PFC (Bioset 1, 2, and 3) were similar to those in entire frontal cortex of developing mice (Additional file 7: Figure S6). Considering that pyramidal neurons are the major population in the cortex [65], maturational abnormality of this cell type would contribute to transcriptomic immaturity in the schizophrenic PFC. Glutaminase is specifically expressed in pyramidal neurons [66] and expression increases during development in human PFC (Additional file 1: Table S8), suggesting that this gene may be a maker for mature pyramidal neurons. The gene was found in all Biosets (Additional file 1: Table S8). This result raises the possibility that pyramidal neurons are also in the immature-like state in the schizophrenic PFC. To examine this question, data on developmental gene expression changes of specific cell types, such as pyramidal neurons, GABAergic neurons other than FS neurons, and microglia, are needed.

Important issues remain to be resolved: how and when the maturational abnormality phenomena emerge in the brains of patients with schizophrenia. Our results indicating the immaturity of multiple cell types in the schizophrenic PFC indicate three possibilities for how such immaturity might occur: failure in cell maturation, reversal of a once-established cell maturation, or recruitment of immature cells. The development and maturation of the brain has long been believed to be a one-way process. However, growing evidence suggests the maturational state is regulated bidirectionally for some cell types in adult brains. Established maturation of granule cells in hippocampal DG can be reversed by chronic treatment with the selective serotonin reuptake inhibitor fluoxetine, which is widely used as an antidepressant [67,68], and by the induction of spontaneous recurrent seizures [69]. Mutation of Shn-2 [11] and SNAP-25 [10] may also reverse the maturational state of DG granule cells postnatally. In the DG of Shn-2 KO mice, 2-week-old animals showed no significant differences between genotypes in the expression of calbindin, a marker of mature granule cells, or calretinin, a marker of immature granule cells. Calbindin expression was decreased and calretinin expression was increased in the DG of 4-week-old Shn-2 KO mice compared with that of wild-type mice, thus indicating that an iDG phenotype emerged during postnatal development [11]. In SNAP-25 KI mice, it was suggested that an iDG phenotype is caused by epileptic seizures that occur after P21–25 [10]. As for FS neurons, fluoxetine treatment induces an immature-like state in the visual cortex [70], basolateral amygdala, hippocampus [71], and the PFC [72] in adulthood. Recently, it was shown that experience could reverse the differentiation state of FS neurons in the adult hippocampus, as monitored by PV expression levels, and the consequent plasticity may influence learning ability [73]. Considering several environmental and genetic factors can induce a reversal in maturational status in these neuron types, it is possible that the immaturity of FS neurons in the schizophrenic PFC represents reversal of the maturational state. Demyelination induced by cuprizone, which could possibly be a partial dematuration of oligodendrocytes, was shown to cause schizophrenia-like symptoms in adult rodents [74], suggesting that reversal of oligodendrocyte maturation may participate in the pathophysiology of schizophrenia.

The third possibility that might explain the transcriptional immaturity of the schizophrenic PFC is adult cortical neurogenesis. New neurons are generated in the cortex of adult rodents and primates under pathological conditions, including ischemia and chemical neurodegeneration [75-78]. Considering that elevated inflammatory conditions have been reported in the cortex of patients with schizophrenia [79], adult neurogenesis might be increased in the schizophrenic cortex [80,81]. A recent study demonstrated that the density of GABAergic interneurons increased in the white matter of the schizophrenic PFC [82], which suggests that new neurons might be generated or recruited in the white matter of patients with schizophrenia. However, neurogenesis is upregulated by less than 1% of the total neuron count in the adult cortex under pathological conditions, such as focal or global ischemia, cortical tissue aspiration, or a laser-induced lesion [83], suggesting that newly generated neurons can hardly account for the transcriptional immaturity of the schizophrenic PFC, even under pathological conditions. Although cortical adult neurogenesis cannot be excluded as a possible contributory factor, it might not be a major factor.

Some studies have addressed the issue of when the maturational abnormality phenomena emerge in the schizophrenic PFC. If the phenomena reflect the consequences of illness chronicity, the magnitude of the alterations would be expected to correlate with illness duration. However, it has been shown that neither illness duration nor age explain the expression levels of some genes related to GABAergic neurons, which are decreased in the schizophrenic PFC, suggesting that the gene expression changes in schizophrenia are not a consequence of illness chronicity [84]. It has also been shown that normal age-related decreases in expression of genes related to central nervous system developmental systems do not occur in patients with schizophrenia during the aging process [7], suggesting that disturbances in gene regulatory mechanisms appear before clinical onset or at an early stage of clinical illness. In the present study, we compared data sets from the DLFC of patients with schizophrenia, who were grouped according to age (defined by Narayan *et al.*[32]), to the two data sets from the developing DLFC (developmental data sets in Figure 1a and b). We found the highest overlap in *P*-values in comparisons with the short-DOI schizophrenia group (Additional file 1: Table S2). Gene expression patterns representing a juvenile-like phenotype are more likely to be associated with younger patients than with older patients. Thus, the altered gene expression that causes abnormalities in neural maturation in schizophrenia seems to emerge during postnatal developmental stages prodromally or concomitantly with clinical onset.

Shn-2 KO mice were previously described as showing multiple schizophrenia-like phenotypes at molecular, anatomical, electrophysiological, and behavioral levels [11]. The transcriptome pattern in the MFC of the Shn-2 KO mice, which we found to be similar to that of human infant MFC in the present study, is also highly similar to that from postmortem patients with schizophrenia [11]. In the present study, we found that genes related to FS neuron development, rather than oligodendrocyte or astrocytes development, showed significant overlap with genes that are commonly up- or downregulated in the MFC of Shn-2 KO mice and human infants (Figure 4). Although the altered gene-expression signals linked to the development of FS neurons may have been derived from maturational abnormalities in other cell types, pseudo-immaturity of FS neurons specifically has been suggested by findings in the MFC of Shn-2 KO mice. We previously reported a decrease in the number of PV-expressing neurons in the MFC of Shn-2 KO mice [11]. Expression of PV in FS neurons in the immature brain was quite low (only one-sixtieth of the adult level) [37], suggesting that the expression of PV could be undetectable in immature FS neurons. Thus, the decrease in the number of PV-expressing neurons in the MFC of Shn-2 KO mice can be at least partly explained by the immaturity of FS neurons.

The results of postmortem analyses often can be confounded by the effects of medications. However, in the genetically engineered rodent model, we can exclude the effect of drugs and control other environmental factors that are not study variables. Therefore, results showing a significant similarity in gene expression patterns between the human developing MFC and the Shn-2 KO mouse MFC suggest that the transcriptional immaturity observed in the schizophrenic PFC may not be due to medication. We also showed that the PFC of rodents treated with antipsychotics exhibited no apparent similarities with the human developing PFC, except for a comparison between olanzapine-treated rodent PFC and the human developing MFC (Additional file 5: Figure S4). These results suggest that medication effect may not be a major contributing factor to the transcriptional immaturity found in the schizophrenic PFC. Other studies also suggest that a decreased expression of GABAergic neuron-related genes in the schizophrenic PFC, including PV, seems unrelated to confounding factors such as medication or substance use [25,84,85].

To date, many hypotheses have been developed to explain the mechanisms of schizophrenia. Immaturity of the PFC seems to be consistent with several major hypotheses, such as the neurodevelopmental [9,86], oligodendrocyte [48,87], and inflammation hypotheses [79,88]. The immaturity of FS neurons literally reflects a neurodevelopmental problem. Dysfunction of oligodendrocytes characterized by demyelination could be accounted for by immaturity of oligodendrocytes, as discussed above. Pathway enrichment analyses showed that enrichment of inflammation-related pathways is likely to be accompanied by a juvenile-like PFC phenotype (Additional file 1: Table S6, S7). Activation of the NADPH-oxidase/interleukin-6 (IL-6) pathway, which is known to play an important role in inflammatory processes, could increase superoxide production in the brain and induce a reversible loss of PV-positive cells in adulthood [89]. In a previous study that used Next Generation Sequencing Expression, IL-6 mRNA was increased in the PFC of patients with schizophrenia, suggesting an increase in inflammation in the schizophrenic PFC [90]. Similarly, oxidative stress increased significantly in the schizophrenic PFC when compared with controls [91,92]. These findings suggest that neuronal pseudo-immaturity in the PFC could be induced by brain inflammation, followed by PFC dysfunction, such as deficits in attention, working memory, and executive functions, which are symptoms of schizophrenia. Together, these findings could be a link between inflammatory conditions and evidence of neuronal immaturity. Enrichments in energy metabolism- and mitochondria-related pathways also were found for genes representing a juvenile-like PFC phenotype (Additional file 1: Table S6, S7, S9). Energy metabolism mediated by mitochondria plays an important role in the development and maintenance of mammalian brains [93]. Because FS neurons have high metabolic demands and show dramatic upregulation of energy related genes during development, it has been hypothesized that defects in energy metabolism genes impair FS neuron development [5]. Thus, enrichments in energy metabolism-related pathways are likely related to abnormalities in cell maturational status, especially in the FS neurons examined in this study. Taken together, these data show that a transcriptional immaturity in the PFC can be considered an endophenotype of schizophrenia, which is consistent with several etiological hypotheses.

Collectively, our results demonstrate that the genome-wide expression profile of the schizophrenic PFC resembles that of the juvenile PFC, which could be due to immaturity in multiple cell types, including FS neurons, astrocytes, and oligodendrocytes. Given that the pseudo-immature cells, especially FS neurons, are not actually lost or absent from the schizophrenic PFC, attempts to restart the normal maturation process could be a potential therapeutic strategy. Considering that adult Shn-2 KO mice also have a juvenile-like PFC, treatments that induce PFC maturation in the mouse model might be candidate therapies for schizophrenia. For example, chronic administration of anti-inflammatory drugs lessened the immaturity of the DG granule cells and some behavioral abnormalities in Shn-2 KO mice [11]. Further investigation of transcriptional immaturity in the PFC as a factor in the precipitation of, as well as recovery from, episodes of schizophrenia would facilitate study of the disorder.

## Conclusions

The present study demonstrates that the schizophrenic PFC resembles the juvenile PFC with respect to transcriptome-wide gene expression profiles. Our results provide evidence that transcriptomic immaturity of PFC may be an endophenotype of schizophrenia.

## Methods

### Data and data processing

Fifteen publicly available microarray data sets were used in this study (Table 1): four data sets on the developing human cortices (GSE13564 [31], GSE25219 [33], GSE11512 [34], GSE37721 [94]), five on the schizophrenic cortices (GSE21138 [32], GSE17612 [36], GSE12649 [35], GSE53987, GSE21935 [95]), two on developing mouse cortex or cells (GSE17806 [37], GSE9566 [38], GSE4675 [96]), one on the medial frontal cortex (MFC) of the schizophrenia mouse model Shn-2 KO mice (GSE42775 [11]), and two on the frontal cortices of rodents treated with antipsychotic drug (GSE45229 [97], GSE2547 [98]). Patient demographics have been previously described in detail [32,35,36,95]. In the study by Narayan *et al*. (GSE21138) [32], schizophrenic subjects were divided into three subgroups according to their duration of illness (DOI), which included a short-DOI, intermediate-DOI, and long-DOI. The mean ages of these subgroups were 26.1 ± 2.05 (short-DOI), 41.9 ± 3.44 (intermediate-DOI), and 65.3 ± 2.91 (long-DOI) years. The control groups were age-matched to their corresponding experimental subgroup and had mean ages of 28.8 ± 2.55, 41.8 ± 2.47, and 64.6 ± 3.26 years. In the study by Maycox *et al*. (GSE17612) [36], the mean ages of schizophrenic and control subjects were 73.3 ± 15.2 and 69.0 ± 21.6 years, respectively. In the study by Iwamoto *et al*. (GSE12649) [35], the mean ages of schizophrenic and control subjects were 42.6 ± 8.5 and 44.1 ± 7.7 years, respectively. In the study by Lanz *et al*. (GSE53987), the mean ages of schizophrenic and control subjects were 46.0 ± 8.6 and 48.1 ± 7.6 years, respectively. Regarding medications, most patients chosen by Narayan *et al*. [32], Maycox *et al.*[36], and Iwamoto *et al.*[35] had been treated with typical antipsychotic drugs. Information regarding medications in the study by Lanz *et al.* is not yet publicly available.

For the analyses examining the effects of antipsychotic treatment on transcriptional immaturity, we used microarray data from antipsychotic-treated rodent brains, which were imported to NextBio by the experimenters. Microarray data from the frontal cortex of mice treated with haloperidol (0.3 or 1 mg/kg/day, N = 4), quetiapine (10 or 100 mg/kg/day, N = 4), or a vehicle (N = 4) for 21 days were obtained from the study published by Kondo *et al*. [97] (GSE45229). Microarray data from the frontal cortex of rats treated with olanzapine (2 mg/kg/day, N = 20) or saline (N = 20) for 21 days were obtained from the study published by Fatemi *et al*. [98] (GSE2547).

All microarray data sets were analyzed using NextBio ( http://www.nextbio.com; Cupertino, CA, USA), a database of microarray experiments. NextBio is a repository of analyzed microarray data sets that allows investigators to search results and expression profiles. The data registered in NextBio goes through rounds of preprocessing, quality control, and curation. Quality assessment methods are employed to review sample-level and dataset-level integrity, which includes reviews of pre- and post-normalization boxplots, missing value counts, and *P*-value histograms (after statistical testing) with false-discovery rate (FDR) analysis to determine whether the number of significantly changing genes is greater than that expected by chance. Other processing of microarray data is by MAS5 (Affymetrix) [99]. Genes with a *P*-value < 0.05 (without correction for multiple testing) and an absolute fold change >1.2 were used in the data set of differentially expressed genes. This is typically the lowest sensitivity threshold used with commercial microarray platforms and the default criterion used in analyses with NextBio. We used Affymetrix GeneChip data selected from the GSE25219 [33], GSE53987, GSE17806 [37], and GSE9566 [38] series, which were downloaded from the NCBI GEO database. Data was pre-processed with Affymetrix Expression Console software using the robust multi-array average algorithm (RMA). Expression values (on a log base-2 scale) were used to calculate fold changes and *P*-values between 2 conditions: young–adults and normal–patients (Table 1). Fold changes were calculated by dividing the young/disease value by the adult/normal value. In the supplementary tables, fold change values are converted into the negative reciprocal, or -1/(fold change), if the fold change is less than 1. Genes with |fold change| >1.2 (GSE25219 and GSE53987) or >1.5 (GSE17806 and GSE9566) and a *t*-test *P*-value <0.05 were imported to NextBio according to the manufacturer’s instructions. Regarding GSE13564 [31], GSE11512 [34], GSE37721 [94], GSE21138 [32], GSE17612 [36], GSE12649 [35], GSE21935 [95], GSE45229 [97], and GSE2547 [98], we used the data imported into NextBio by the experimenters. Data for GSE42775 were previous experimental results from our laboratory [11] and were imported into NextBio. We compared the signatures in two given gene sets using NextBio. Overlap between gene sets was calculated using the Running Fisher test [99]. This non-parametric rank-based statistical method developed by NextBio enabled us to statistically assess pairwise correlations between any two datasets. Directional information (up- or downregulation) for each gene also was incorporated in the analysis to assess the similarities between datasets.

To enable comparison across different arrays, orthologs were identified for each pair of organisms [99]. Ortholog identification was based on information obtained from Mouse Genome Informatics (MGI) at Jackson Lab ( http://www.informatics.jax.org), HomoloGene at NCBI ( http://www.ncbi.nlm.nih.gov), and Ensembl ( http://www.ensembl.org). The gene overlap *P*-value calculated by NextBio indicates a statistically significant association between two given gene sets. The detailed methods for comparison of data sets are given in Additional file 8. The significance level of the *P*-value for overlap between data sets was corrected for the number of combinations of data sets (enumerated in Additional file 1: Table S27) using the Bonferroni method. The level of significance for overlap in each pair of expression-change directions was also corrected for the number of possible situations (up, up; down, down; up, down; down, up) using Bonferroni method (*P* < 0.0125 = 0.05/4).

### Statistical analysis

Pearson’s chi-square test was applied to the number of genes with a “positive correlation” or a “negative correlation,” which were compared with the expected frequency of 50%. Significance was defined as a *P* < 0.05.

### Pathway analysis

The DAVID functional annotation clustering tool (david.abcc.ncifcrf.gov) was used to assess gene lists for enrichment in biological themes [100]. The gene list (all genes in Biosets 1–3 [Figure 2]) was processed through DAVID using the default feature listings and algorithm settings, with the Affymetrix 3′ IVT (Human Genome U133 Plus 2) as a background. *P*-values from Fisher’s exact tests of enrichment proportion were Benjamini-adjusted for multiple testing in DAVID (Additional file 1: Table S6). We also analyzed for pathway enrichment to find biogroups for which our gene lists (Bioset 1, 2, and 3) were highly enriched using NextBio software. We identified the top 30 biogroups for enrichment score based on the input gene signature (Additional file 1: Table S7).

## Abbreviations

BA: Brodmann’s area; DG: Dentate gyrus; DLFC: Dorsolateral prefrontal cortex; FS neurons: Fast-spiking neurons; GABA: Gamma-aminobutyric acid; MFC: Medial prefrontal cortex; PFC: Prefrontal cortex; PNNs: Perineuronal nets; Shn-2: Schnurri-2; STC: Superior temporal cortex.

## Competing interests

Tsuyoshi Miyakawa is an advisor/consultant for Astellas Pharma Inc. The other authors declare no conflict of interest.

## Authors’ contributions

HH and TM conceived the study. TM led the project. HH performed the bioinformatic analyses. HH, KO, KT and TM co-wrote the paper. All authors read and approve the manuscript.

## Supplementary Material

Additional file 1 Supplementary Tables S1-S27. Click here for file

Additional file 2: Figure S1 Comparison of gene expression patterns between the developing and adult schizophrenic DLFC. The gene expression pattern in the DLFC (BA46) of patients with schizophrenia (patients compared with controls; GSE53987 **[a, b]** or GSE12649 **[c, d]**) was compared with that in the DLFC (BA46) of normal infants (GSE11512, infants, 0.1–0.3 years, compared with adults 20–49 years). The Venn diagrams illustrate the overlap in transcriptome-wide gene expression changes in the DLFC of patients with schizophrenia (patients compared with controls) and normal infants (infants compared with adults) **(a, c)**. Bar graphs illustrate the *P*-values of overlaps of genes upregulated (*red arrows*) or downregulated (*blue arrows*) by each condition, between the two conditions **(b, d)**. Click here for file

Additional file 3: Figure S2 Comparison of gene expression patterns between the schizophrenic MFC and the normal infant MFC as compared to elderly adults. **(a)** The gene expression pattern in the MFC (BA10) of patients with schizophrenia (GSE17612, patients [73.3 ± 15.2 years] compared with controls [69.0 ± 21.6 years]) was compared with that in the MFC (BA24, 32, 33) of normal infants (GSE25219, infants, 1–5 years, compared with adults over 60 years). **(b)** Venn diagrams illustrating the overlap in transcriptome-wide gene expression changes in the MFC of patients with schizophrenia (patients compared with controls) and normal infants (infants, 1–5 years, compared with adults over 60 years). **(c)** Bar graphs illustrate the *P*-values of overlaps of genes upregulated (*red arrows*) or downregulated (*blue arrows*) by each condition, between the two conditions. Click here for file

Additional file 4: Figure S3 Transcripts overlapped across three Biosets. The Venn diagram illustrates the extent of overlap across Biosets. Among the 981 probes found in any of the three Biosets, 22 were shared with those Biosets. The complete gene list is provided in Additional file 1: Table S8. Click here for file

Additional file 5: Figure S4 Comparison of gene expression patterns between the human developing PFC and antipsychotic-treated rodent frontal cortex. **(a–e)** The gene expression pattern in the DLFC (BA46) of normal infants (GSE13564, infants <2 years, compared to adults 20–49 years) was compared with that in the frontal cortex of rodents treated with haloperidol (GSE45229) **(a, b)**, quetiapine (GSE45229) **(c, d)**, or olanzapine (GSE2547) **(e)**. **(f–j)** The gene expression pattern in the DLFC (BA9 and 46) of normal infants (GSE25219, infants, 1–5 years, compared with adults 20–39 years) was compared with that in the frontal cortex of rodents treated with haloperidol (GSE45229) **(f, g)**, quetiapine (GSE45229) **(h, i)**, or olanzapine (GSE2547) **(j)**. **(k–o)** The gene expression pattern in the MFC (BA24, 32, 33) of normal infants (GSE25219, infants, 1–5 years, compared with adults 20–39 years) was compared with that in the frontal cortex of rodents treated with haloperidol (GSE45229) **(k, l)**, quetiapine (GSE45229) **(m, n)**, or olanzapine (GSE2547) **(o)**. Venn diagrams illustrate the overlap in transcriptome-wide gene expression changes between conditions. Bar graphs illustrate the *P*-values of overlaps of genes upregulated (*red arrows*) or downregulated (*blue arrows*) by each condition, between the two conditions. N/A (not applicable) means that the overlap *P*-values exceeded the NextBio database cutoff, which is the default criterion used in analyses by NextBio (a *P*-value of approximately 0.6). Click here for file

Additional file 6: Figure S5 Comparison of gene expression patterns between the developing and schizophrenic STC. The gene expression pattern in the STC (BA22) of patients with schizophrenia (GSE21935, patients [72.2 ± 16.9 years] compared with controls [67.7 ± 22.2 years]) was compared with that in the STC of normal infants (GSE25219, infants, 1–5 years, compared with adults 20–39 years) **(a–c)**, or with that in the STC of normal infants (GSE37721, infants, 2–6 years, compared with adults 24–48 years) **(d–f)**. **(b, e)** Venn diagrams illustrating the overlap in transcriptome-wide gene expression changes in the STC of patients with schizophrenia (patients compared with controls) and normal infants (infants compared with adults). **(c, f)** Bar graphs illustrate the *P*-values of overlaps of genes upregulated (*red arrows*) or downregulated (*blue arrows*) by each condition, between the two conditions. Click here for file

Additional file 7: Figure S6 Comparison of gene expression patterns between each Bioset and mouse whole frontal cortex development. Genes showing the same directional changes in expression between the normal developing and adult schizophrenic PFC (Bioset1 **[a, b]**, Bioset2 **[c, d]**, Bioset3 **[e, f]**, as shown in Figure 2) were compared to those obtained from developmental experiments on mouse whole frontal cortex (GSE4675). **(a, c, e)** Venn diagrams illustrate the overlap in transcriptome-wide gene expression changes between two conditions. **(b, d, f)** Bar graphs illustrate the *P*-values of overlaps of genes upregulated (*red arrows*) or downregulated (*blue arrows*) by each condition, between the two conditions. Click here for file

Additional file 8 Supplementary methods. Click here for file
