# Supplementary figures and images for: Transcriptomic evidence for immaturity of the prefrontal cortex in patients with schizophrenia
Source: Mol Brain. 2014 May 29;7:41. doi: 10.1186/1756-6606-7-41 (PMC4066280; doi:10.1186/1756-6606-7-41)

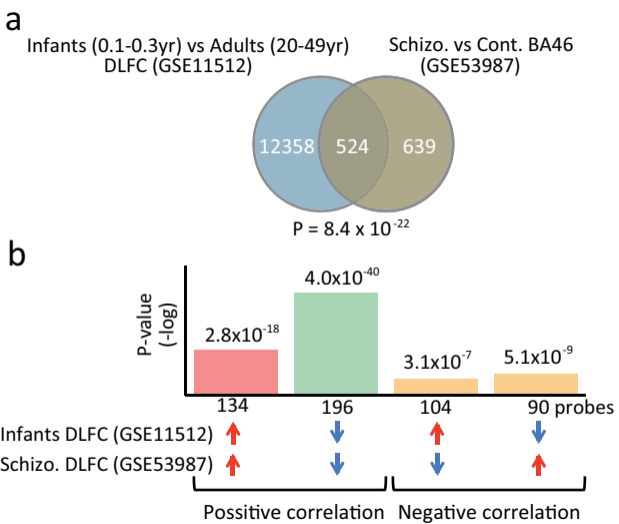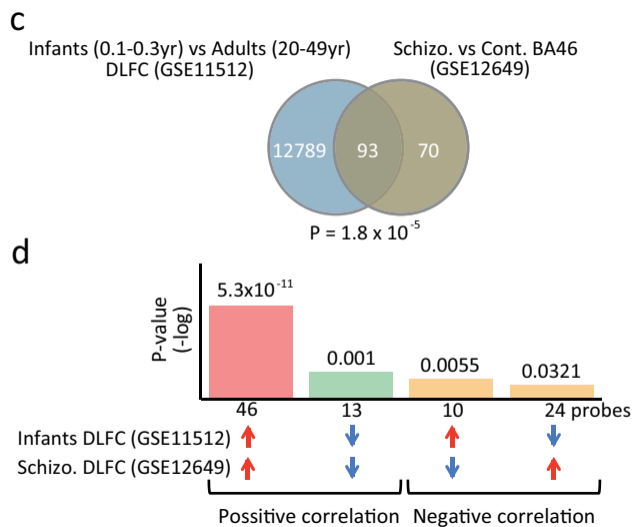

Supplement: Additional file 2: Figure S1 — Comparison of gene expression patterns between the developing and adult schizophrenic DLFC. The gene expression pattern in the DLFC (BA46) of patients with schizophrenia (patients compared with controls; GSE53987 [a, b] or GSE12649 [c, d]) was compared with that in the DLFC (BA46) of normal infants (GSE11512, infants, 0.1–0.3 years, compared with adults 20–49 years). The Venn diagrams illustrate the overlap in transcriptome-wide gene expression changes in the DLFC of patients with schizophrenia (patients compared with controls) and normal infants (infants compared with adults) (a, c). Bar graphs illustrate the P-values of overlaps of genes upregulated (red arrows) or downregulated (blue arrows) by each condition, between the two conditions (b, d). [file 1756-6606-7-41-S2.pdf]

a

Development of MFC (GSE25219)

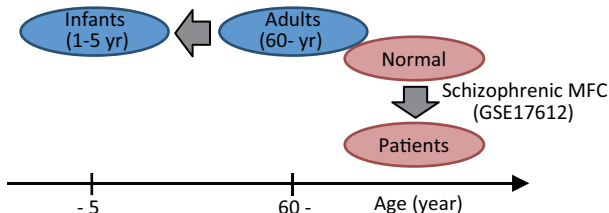

b

Infants (1-5yr) vs Adults (60yr)  
MFC (GSE25219)Schizo. vs Cont. BA10  
(GSE17612)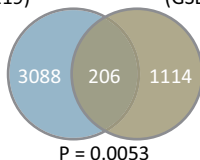

c

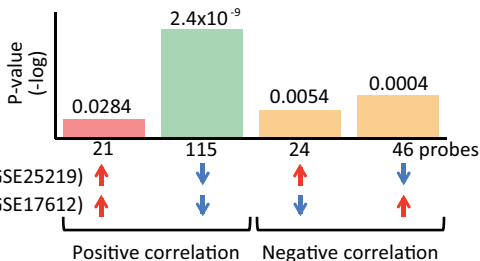

Supplement: Additional file 3: Figure S2 — Comparison of gene expression patterns between the schizophrenic MFC and the normal infant MFC as compared to elderly adults. (a) The gene expression pattern in the MFC (BA10) of patients with schizophrenia (GSE17612, patients [73.3 ± 15.2 years] compared with controls [69.0 ± 21.6 years]) was compared with that in the MFC (BA24, 32, 33) of normal infants (GSE25219, infants, 1–5 years, compared with adults over 60 years). (b) Venn diagrams illustrating the overlap in transcriptome-wide gene expression changes in the MFC of patients with schizophrenia (patients compared with controls) and normal infants (infants, 1–5 years, compared with adults over 60 years). (c) Bar graphs illustrate the P-values of overlaps of genes upregulated (red arrows) or downregulated (blue arrows) by each condition, between the two conditions. [file 1756-6606-7-41-S3.pdf]

Total 981 probes

BioSet1

295

239

5

22

263

139

18

BioSet2

BioSet3

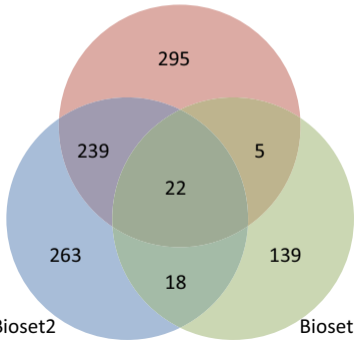

Supplement: Additional file 4: Figure S3 — Transcripts overlapped across three Biosets. The Venn diagram illustrates the extent of overlap across Biosets. Among the 981 probes found in any of the three Biosets, 22 were shared with those Biosets. The complete gene list is provided in Additional file 1: Table S8. [file 1756-6606-7-41-S4.pdf]

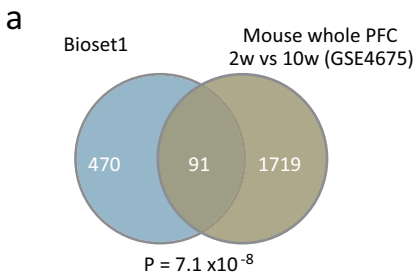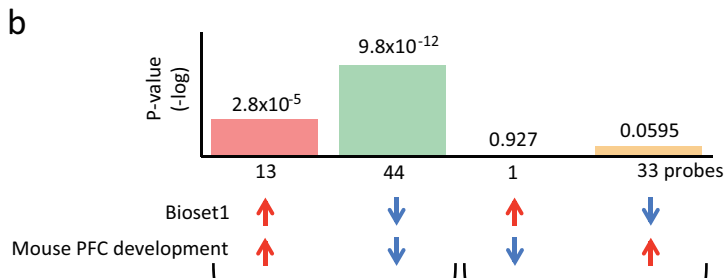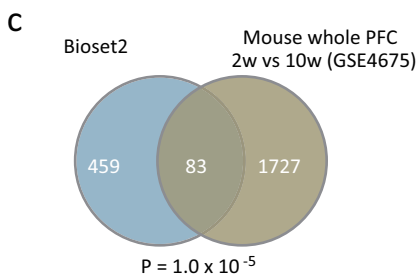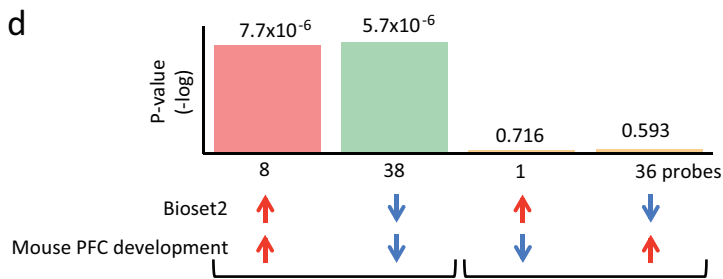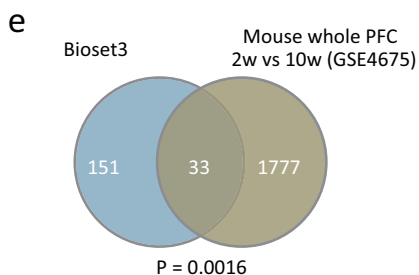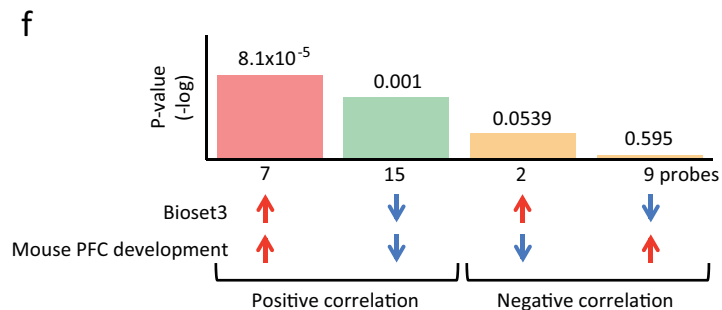

Supplement: Additional file 7: Figure S6 — Comparison of gene expression patterns between each Bioset and mouse whole frontal cortex development. Genes showing the same directional changes in expression between the normal developing and adult schizophrenic PFC (Bioset1 [a, b], Bioset2 [c, d], Bioset3 [e, f], as shown in Figure 2) were compared to those obtained from developmental experiments on mouse whole frontal cortex (GSE4675). (a, c, e) Venn diagrams illustrate the overlap in transcriptome-wide gene expression changes between two conditions. (b, d, f) Bar graphs illustrate the P-values of overlaps of genes upregulated (red arrows) or downregulated (blue arrows) by each condition, between the two conditions. [file 1756-6606-7-41-S7.pdf]
