# Supplementary material for: Transcriptomic evidence for immaturity of the prefrontal cortex in patients with schizophrenia
Source: Mol Brain. 2014 May 29;7:41. doi: 10.1186/1756-6606-7-41 (PMC4066280; doi:10.1186/1756-6606-7-41)
Supplement: Additional file 5: Figure S4 — Comparison of gene expression patterns between the human developing PFC and antipsychotic-treated rodent frontal cortex. (a–e) The gene expression pattern in the DLFC (BA46) of normal infants (GSE13564, infants <2 years, compared to adults 20–49 years) was compared with that in the frontal cortex of rodents treated with haloperidol (GSE45229) (a, b), quetiapine (GSE45229) (c, d), or olanzapine (GSE2547) (e). (f–j) The gene expression pattern in the DLFC (BA9 and 46) of normal infants (GSE25219, infants, 1–5 years, compared with adults 20–39 years) was compared with that in the frontal cortex of rodents treated with haloperidol (GSE45229) (f, g), quetiapine (GSE45229) (h, i), or olanzapine (GSE2547) (j). (k–o) The gene expression pattern in the MFC (BA24, 32, 33) of normal infants (GSE25219, infants, 1–5 years, compared with adults 20–39 years) was compared with that in the frontal cortex of rodents treated with haloperidol (GSE45229) (k, l), quetiapine (GSE45229) (m, n), or olanzapine (GSE2547) (o). Venn diagrams illustrate the overlap in transcriptome-wide gene expression changes between conditions. Bar graphs illustrate the P-values of overlaps of genes upregulated (red arrows) or downregulated (blue arrows) by each condition, between the two conditions. N/A (not applicable) means that the overlap P-values exceeded the NextBio database cutoff, which is the default criterion used in analyses by NextBio (a P-value of approximately 0.6). [file 1756-6606-7-41-S5.pdf]

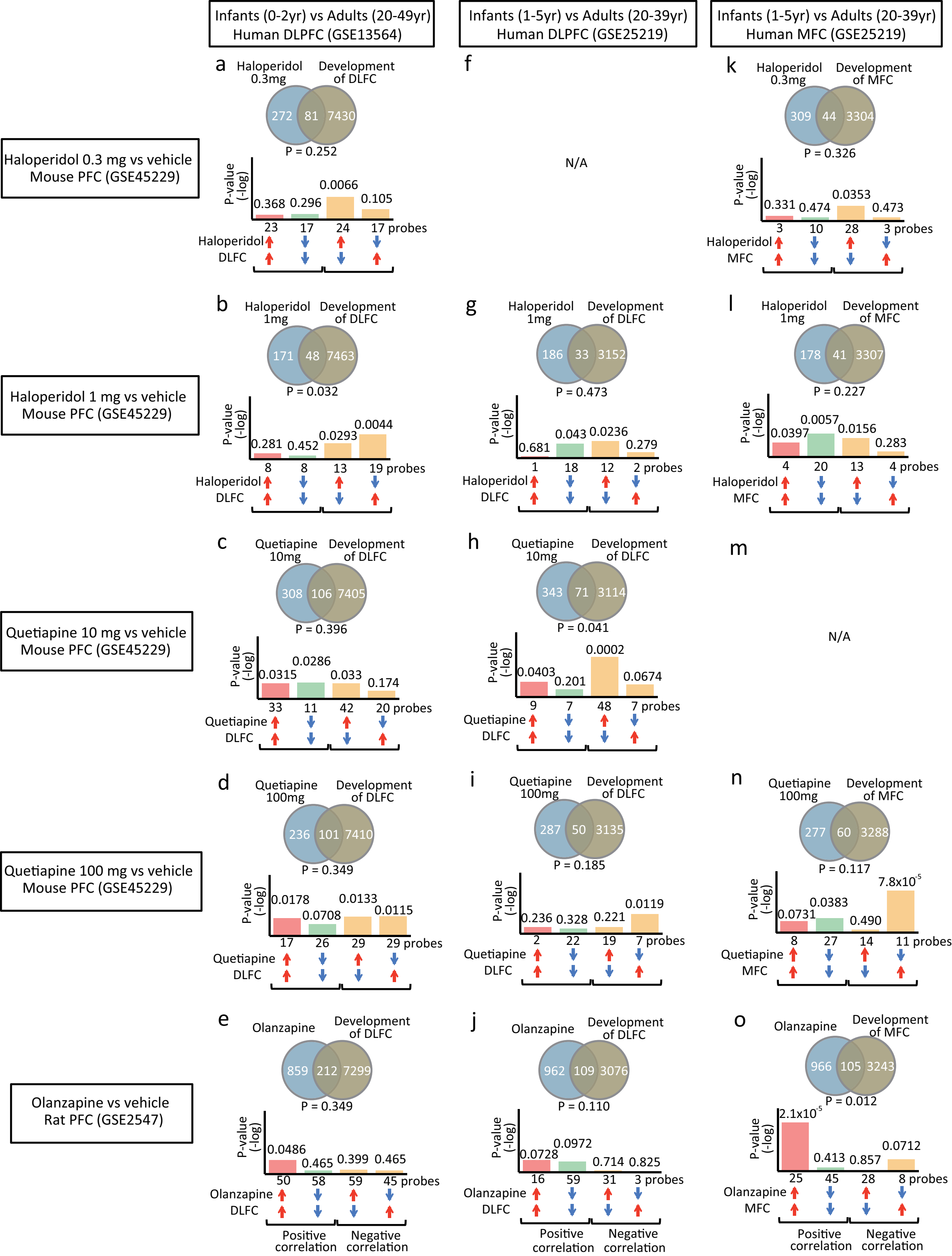

Infants (0-2yr) vs Adults (20-49yr) Human DLPFC (GSE13564)
Infants (1-5yr) vs Adults (20-39yr) Human DLPFC (GSE25219)
Infants (1-5yr) vs Adults (20-39yr) Human MFC (GSE25219)
Haloperidol 0.3 mg vs vehicle Mouse PFC (GSE45229)
Haloperidol 1 mg vs vehicle Mouse PFC (GSE45229)
Quetiapine 10 mg vs vehicle Mouse PFC (GSE45229)
Quetiapine 100 mg vs vehicle Mouse PFC (GSE45229)
Olanzapine vs vehicle Rat PFC (GSE2547)
a
Haloperidol 0.3mg
Development of DLFC
272
81
7430
P = 0.252
P-value (-log)
0.368 0.296 0.0066 0.105
23 17 24 17 probes
Haloperidol
DLFC
f
N/A
k
Haloperidol 0.3mg
Development of MFC
309
44
3304
P = 0.326
0.331 0.474 0.0353 0.473
3 10 28 3 probes
Haloperidol
MFC
b
Haloperidol 1mg
Development of DLFC
171
48
7463
P = 0.032
0.281 0.452 0.0293 0.0044
8 8 13 19 probes
g
Haloperidol 1mg
Development of DLFC
186
33
3152
P = 0.473
0.681 0.043 0.0236 0.279
1 18 12 2 probes
l
Haloperidol 1mg
Development of MFC
178
41
3307
P = 0.227
0.0397 0.0057 0.0156 0.283
4 20 13 4 probes
c
Quetiapine 10mg
Development of DLFC
308
106
7405
P = 0.396
0.0315 0.0286 0.033 0.174
33 11 42 20 probes
Quetiapine
DLFC
h
Quetiapine 10mg
Development of DLFC
343
71
3114
P = 0.041
0.0403 0.201 0.0002 0.0674
9 7 48 7 probes
m
N/A
d
Quetiapine 100mg
Development of DLFC
236
101
7410
P = 0.349
0.0178 0.0708 0.0133 0.0115
17 26 29 29 probes
i
Quetiapine 100mg
Development of DLFC
287
50
3135
P = 0.185
0.236 0.328 0.221 0.0119
2 22 19 7 probes
n
Quetiapine 100mg
Development of MFC
277
60
3288
P = 0.117
0.0731 0.0383 0.490 7.8x10-5
8 27 14 11 probes
Quetiapine
MFC
e
Olanzapine
Development of DLFC
859
212
7299
P = 0.349
0.0486 0.465 0.399 0.465
50 58 59 45 probes
Olanzapine
DLFC
Positive correlation
Negative correlation
j
Olanzapine
Development of DLFC
962
109
3076
P = 0.110
0.0728 0.0972 0.714 0.825
16 59 31 3 probes
o
Olanzapine
Development of MFC
966
105
3243
P = 0.012
2.1x10-5 0.413 0.857 0.0712
25 45 28 8 probes
Olanzapine
MFC
