# Supplementary material for: Transcriptomic evidence for immaturity of the prefrontal cortex in patients with schizophrenia
Source: Mol Brain. 2014 May 29;7:41. doi: 10.1186/1756-6606-7-41 (PMC4066280; doi:10.1186/1756-6606-7-41)
Supplement: Additional file 6: Figure S5 — Comparison of gene expression patterns between the developing and schizophrenic STC. The gene expression pattern in the STC (BA22) of patients with schizophrenia (GSE21935, patients [72.2 ± 16.9 years] compared with controls [67.7 ± 22.2 years]) was compared with that in the STC of normal infants (GSE25219, infants, 1–5 years, compared with adults 20–39 years) (a–c), or with that in the STC of normal infants (GSE37721, infants, 2–6 years, compared with adults 24–48 years) (d–f). (b, e) Venn diagrams illustrating the overlap in transcriptome-wide gene expression changes in the STC of patients with schizophrenia (patients compared with controls) and normal infants (infants compared with adults). (c, f) Bar graphs illustrate the P-values of overlaps of genes upregulated (red arrows) or downregulated (blue arrows) by each condition, between the two conditions. [file 1756-6606-7-41-S6.pdf]

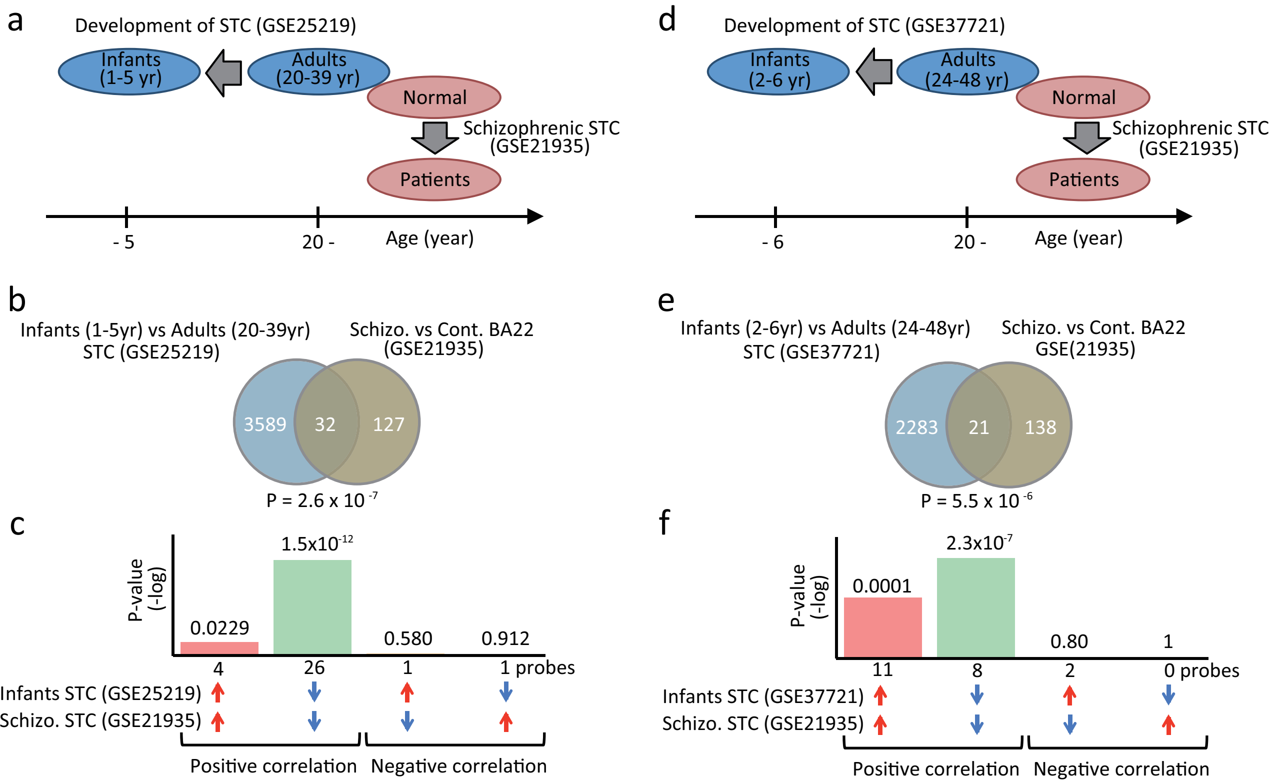
a
Development of STC (GSE25219)
Infants (1-5 yr)
Adults (20-39 yr)
Normal
Schizophrenic STC (GSE21935)
Patients
- 5
20 -
Age (year)
d
Development of STC (GSE37721)
Infants (2-6 yr)
Adults (24-48 yr)
Normal
Schizophrenic STC (GSE21935)
Patients
- 6
20 -
Age (year)
b
Infants (1-5yr) vs Adults (20-39yr) STC (GSE25219)
Schizo. vs Cont. BA22 (GSE21935)
3589
32
127
P = 2.6 x 10 -7
e
Infants (2-6yr) vs Adults (24-48yr) STC (GSE37721)
Schizo. vs Cont. BA22 GSE(21935)
2283
21
138
P = 5.5 x 10 -6
c
P-value (-log)
1.5x10-12
0.0229
0.580
0.912
4
26
1
1 probes
Infants STC (GSE25219)
Schizo. STC (GSE21935)
Positive correlation
Negative correlation
f
P-value (-log)
2.3x10-7
0.0001
0.80
1
11
8
2
0 probes
Infants STC (GSE37721)
Schizo. STC (GSE21935)
Positive correlation
Negative correlation
