# Supplementary material for: Transcriptomic evidence for immaturity of the prefrontal cortex in patients with schizophrenia
Source: Mol Brain. 2014 May 29;7:41. doi: 10.1186/1756-6606-7-41 (PMC4066280; doi:10.1186/1756-6606-7-41)
Supplement: Additional file 8 — Supplementary methods. [file 1756-6606-7-41-S8.doc]

**Supplementary methods**

**Computing overlap p-value of gene expression patterns from different datasets**

NextBio compares the signatures in publicly available microarray databasets with a signature provided by the user using a “Running Fisher” algorithm, as previously described [99]. The overlap *P* value, i.e., the direction of the correlation between two given gene signature sets (*b1*, *b2*), and the *P* values between subsets of gene signatures, is calculated as follows:

First, each gene signature set was rank-ordered according to the absolute fold-change value. Upregulated and downregulated genes were denoted by positive and negative signs, respectively, to imply directionality. A directional subset was generated for each direction, such as *b1+*, *b1-*, *b2+*, and *b2-*. Second, all of the subset pairs were identified as *b1Di*, *b2Dj*, where *Di* and *Dj* were the available directions (+ or -) in *b1* and *b2*, respectively. The Running Fisher algorithm was applied to each subset pair. The top ranking genes in the first subset *b1Di* were collected as a group, *G*, and the second subset *b2Dj* was scanned from top to bottom in rank order to identify each rank with a gene matching a member in group *G*. At each matching rank, *K*, the scanned portion of the second subset *b2Dj* consisted of *N* genes, and the overlap between group *G* and *N* genes was defined as *M*. A Fisher’s exact test was performed at rank *K* to evaluate the statistical significance of observing *M* overlaps between a set of size *G* and a set of size *N*, where the set of size *G* comes from platform *P1* and the set of size *N* comes from platform *P2*, given the sizes of *P1* and *P2* as well as the overlap between *P1* and *P2*. At the end of the scan, the best *P* value was retained, and a multiple hypothesis testing correction factor was applied. The negative log of the multiple testing corrected best *P* value (
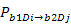
) was a score (
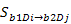
) for the subset pair. Here, the subscript of
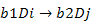
 indicates that *b*1*Di* was the first subset used to define the top genes *G* and *b*2*Dj* was the second subset that is used for the scan.


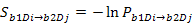
. (1)

Next, the Running Fisher algorithm was performed in the reverse direction. The same procedure in this reverse direction produced another score (
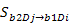
) for the same subset pair. The two scores were averaged to represent the magnitude of the similarity between the two subsets.


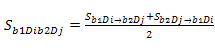
. (2)

The *P* value (*P_b1Dib2Dj_*) between *b1Di* and *b2Dj* was calculated using the following equation:


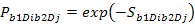
. (3)

A positive sign was assigned to pairwise correlation scores (*S_b1+b2+_* and *S_b1-b2-_*) for a subset pair of the same direction (*b1+b2+*, *b1-b2-*), and a negative sign was assigned to pairwise correlation scores (*S_b1+b2-_* and *S_b1-b2+_*) for a subset pair of opposite directions (*b1+b2-*, *b1-b2+*). Then, the overall score (*S_b1b2_*) between *b1* and *b2* was calculated from the correlation scores (*S_b1+b2+_*, *S_b1-b2-_*, *S_b1+b2-_*, and *S_b1-b2+_*) of subset pairs using the following equation:


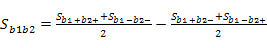
 . (4)

The sign of *S_b1b2_* determined whether the two signatures were positively or negatively correlated. The overall *P* value (*P_b1b2_*) between *b1* and *b2* was calculated using the following equation:


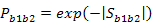
. (5)

This overall *P* value was referred as an overlap *P* value between two gene expression patterns in this paper.
